# Nurses Burnout, Resilience, and Its Association With Socio-Demographic Factors During COVID-19 Pandemic

**DOI:** 10.3389/fpsyt.2021.803506

**Published:** 2022-01-14

**Authors:** Majid Heidari Jamebozorgi, Ali Karamoozian, Tayebe Ilaghinezhad Bardsiri, Hojjat Sheikhbardsiri

**Affiliations:** ^1^Department of Public Health, Sirjan School of Medical Sciences, Sirjan, Iran; ^2^Modeling in Health Research Center, Institute for Futures Studies in Health, Kerman University of Medical Sciences, Kerman, Iran; ^3^Department of Biostatistics and Epidemiology, Kerman University of Medical Sciences, Kerman, Iran; ^4^Department of Neonatal Intensive Care Nursing, Faculty of Nursing, School of Nursing and Midwifery, Sirjan University of Medical Sciences, Sirjan, Iran; ^5^Health in Disasters and Emergencies Research Center, Institute for Futures Studies in Health, Kerman University of Medical Sciences, Kerman, Iran

**Keywords:** burnout, resiliency, nurses, socio-demographic, COVID-19

## Abstract

**Background:**

In the recent pandemic, nurses have faced workload and being exposed to burnout. Resilience helps address work-related psychological problems such as stressful events and burnout. According to the roles of nurses in the healthcare system, we investigated the relationship between resiliency and burnout in nurses.

**Material and Methods:**

In this descriptive analytical cross-sectional study, 364 nurses participated from April to June 2021. Census sampling was used to recruit participants. Maslach burnout inventory (MBI), Connor-Davidson Resiliency Scale (CDRISC), and a demographic check-list were utilized to collect data. Data analysis was done using SPSS version 22. Shapiro-Wilk, Kruskal–Wallis test, Mann–Whitney *U*-test, correlation analysis, and generalized linear model were applied accordingly.

**Results:**

Overall, the findings showed that nurses had severe symptoms of burnout and a moderate level of resilience. The two domains of burnout, emotional exhaustion and personal accomplishment had a significantly negative correlation with resilience (*r* = −0.442, *p* < 0.001 and *r* = −0.351, *p* = 0.03, respectively). Linear regression showed that demographic characteristics (Hospital type, ward type, gender, and overtime) were the major predictors of the 3 sub-categories of burnout. A significant negative correlation was observed between burnout and resilience highlighting the role of resilience in reducing burnout (*P* < 0.05).

**Conclusion:**

In order to help nurses to tackle and endure burnout in pandemic times, there is a need to implement national and local policies to help them accordingly.

## Introduction

With its rapid global spread and concomitant mortality burden, the COVID-19 outbreak represents a global public health issue unseen in the last century. Health systems around the world have encountered unprecedented problems in resourcing a healthcare response as SARS-CoV-2 spreads fast (1–3). Amid the pandemic of COVID-19, burnout is a critical health-care issue which involves healthcare employees in numerous jobs, in particular nurses. With regard to the experience nurses have concerning physical and psychological stressors, they face the phenomenon of occupational burnout. As a result, they are more vulnerable to negative mental health outcomes amidst COVID-19s (4–6).

Accordingly, nurses are one of the most crucial pillars of healthcare organizations during COVID-19, and any flaws will have irreversible implications owing to their critical role in patient care. As a result, it is critical to pay close attention to the factors that influence nurses' performance in this area (7, 8). Burnout among nurses, more than any other factors, jeopardizes patient recovery (9, 10). This issue is a major contributor to decreased productivity, and issues related to mental and physical topics (11). Due to the prevalence of COVID-19 and the heavy workload of nurses in hospitals and health care centers, managers must take heed to the burnout of nurses (12). During the COVID-19 outbreak period, few researches have been conducted on burnout levels among nurses. The study done by Bashirian et al. shows that, nurses experience more burnout in comparison to other health workers during the pandemic of COVID-19 (13).

Physical, emotional, and mental health are all harmed by burnout. Efficient managing of the sources of stress that contribute to burnout is one of the most significant factors in preventing burnout (14). The essential aspects of stress management and burnout prevention are an individual's personality qualities and psychological processes (15). Managing the issues related to pandemics, our knowledge and adaptation to come up with ways to overcome them is essential. Resilience is understood in this way. According to a literature, resilience has been a controlling factor against mental illnesses, including anxiety and depression. Studies suggest that resilience is relevant to health care workers' anxiety levels, in the sense that the more resilient a person is, the better his mental health will be (16, 17).

The concept of psychological resilience has arisen in recent years as a personality feature that protects against burnout. Despite a variety of explanations concentrating on various facets of resilience, which has a complex and teachable structure, the adaptation of a person to major stressful factors such as employment and financial challenges is defined as resilience (18). More resilient people are better equipped to deal with obstacles, uncertainty, and other unpleasant conditions, increasing their capacity to succeed. Nurses who have improved their resilience are better equipped to cope with bad situations, have increased their ability to adapt and achieve, and are likely to have less burnout (19–21).

Several studies on the association between resilience and burnout have been undertaken, but few have included nurses (19, 21–24). Nurse burnout is not just a problem in the workplace or a concern for policymakers; it has become a universal issue. Taking into account the nurse burnout and its relation to resilience are crucial for improving nurses' emotional and physical health, as well as the quality of care delivered by them Therefore, this study aims to investigate the level of burnout and resilience in nurses and to investigate whether there is a link between the level of resilience and burnout in nurses amid the pandemic of corona virus.

## Method

### Design and Participants

This self-reported cross-sectional study was undertaken based on quantitative data in April–June 2021. The study was conducted in two hospitals in southeastern Iran. Participants were recruited from the only COVID-19-designated hospitals. Our inclusion criteria encompassed having a bachelor's degree or higher and work experience more than of 6 months. We excluded participants on account of unwillingness to take part in research and not completed questionnaires. We obtained informed consent and assured them regarding the confidentiality of their information. The reminder messages were sent to the nurses by researchers, so most of the questionnaires (96%) were collected in June.

### Sampling Method and Sample Size

The sample size was calculated using the findings of a former study (25) indicating that the prevalence of burnout among nurses was 30.6%. Therefore, with a *Z* = 1.96 and a *d* = 0.05, a sample size of 326 subjects was obtained. As the calculated sample size was almost equal to the size of the total population of nurses in the study setting, all eligible nurses in the setting were recruited to the study through a census sampling.

### Data Collection

Because of COVID-19 outbreak, data were gathered through online questionnaires via the Porsline application to minimize the COVID-19 transmission risk between researchers and respondents. The survey was sent using Porsline online service system (https://survey.porsline.ir/s/gyAh8hB); the link to the survey was disseminated via social media (Telegram, WhatsApp, and Instagram). The participants gave informed consent before the initiation of the study. To avoid null values, all elements were adjusted as needed. In order to increase the response rate, the participants were reminded to complete the survey every week in April (during the pandemic) and the survey was closed on May 1, 2021. The survey tool consisted of 71 questions. It also included three well-recognized and validated tools. Three parts of the tool had to be completed: demographic characteristics information, the Maslach Burnout Inventory–Human Services Survey (MBI-HSS) questionnaire, and the Connor–Davidson Resilience Scale (CR-RISC) questionnaire.

#### Measurements

##### Socio-Demographic Attributes

The demographic characteristics information cheklist had two different parts. The first part encompassed demographic items (age, sex, marital status, and educational level), and the second part included career traits (years of professional work, type of ward, and overtime hours).

##### The Maslach Burnout Index

The used questionnaire has been developed and validated previously (26). It has 22 questions which divided into 3 sub-categories: emotional exhaustion (EE), depersonalization (DP), and personal accomplishment (PA). In the dimension of EE, eight items were associated with fatigue, being tired, and decreased emotional energy. In the dimension of DP, 6 items relate to the behavior of an individual lacking affection toward those being cared for and served. In the dimension of PA, 8 items identify situations in which a person feels sufficient and successful (10, 27). Each item has a 5-point Likert scale that varies from “never” or 0 to “every day” or 6. Although there is no defined cut-off for the MBI subscales, based on **‘a** previous study (28) which examined the most commonly used raw score threshold. For EE, scores of 27 or higher are classified as high, scores below 16 are low, and scores between 17 and 26 are moderate. For DP, a score of 13 or higher is classified as high, a score below 6 is low, and a score of 7 to 12 is moderate. For PA, a score of 31 or below is considered low, a score above 39 is high, and a score of 32 to 38 is moderate. Low, high, and moderate burnout levels are represented by scores ≤53, ≥79, and 54–78. To determine burnout, scores for the 3 sub-dimensions were evaluated separately. In this study, Cronbach's alpha coefficients for EE, DP, and PA were 0.89, 0.84, and 0.79, respectively.

##### Connor–Davidson Resilience Scale−25

This scale has 25 items over three sub-categories (tenacity, strength, and optimism) that evaluate resilience or flexibility to change and cope with adversity. A 5-point Likert scale was used (0 = not true at all, 4 = always true). The total score ranges from 0 to 100, with higher scores indicating higher degrees of resilience. In our study, the Cronbach alpha coefficients of resilience and its three sub-categories were 0.89, 0.81, 0.79, and 0.76, respectively (29).

#### Data Analysis

In order to analyze data, we used SPSS software (v. 22.0). Mean and standard deviation (Mean ± SD) were calculated for numerical variables, while absolute and relative frequencies were calculated for categorical variables. We tested normality by using the Shapiro-Wilk test. In order to investigate the relationship between the variables in the present study, Mann-Whitney, Kruskal Wallis, Spearman correlation, and generalized linear regression models were used. Also, in order to determine the significance of the variables, a significance level of 5% was used and those variables whose *p*-value was estimated to be <0.05 were recognized as influential variables in the model.

## Results

The demographic characteristics as well as the MBI and CDRISC mean scores in each subscale of participants are presented in Table 1. A total of 378 nurses in the study setting, 364 nurses (113 from public hospital and 251 from social security hospital) completely answered the study questionnaires (response rate: 96%). Most participants worked in second line departments (59.30%), were female (84.1%), held an undergraduate degree in nursing (90%), worked with a range from overtime between 60 and 100 h per month (44.5%), the means of their age and work experience were 37.20 ± 6.38 and 15.12 ± 6.52 years, respectively.

**Table 1:** The compare of resilience and burnout mean score of participant's base of demographic variables.

**Variable**	**Resilience**				**Burnout**			
	**Frequency (%)**	**Mean ±SD**	**Median (IQR)**	***p*** **-value**	**Frequency (%)**	**Mean ±SD**	**Median (IQR)**	***p*** **-value**
**Hospital**				0.17				**0.003**
Gharazi	113 (31.00)	55.94 ± 8.91	54 (10.50)		113 (31.00)	71.39 ± 11.73	70 (11)	
Emam Reza	251 (69.00)	57.37 ± 11.89	56 (11)		251 (69.00)	76.45 ± 14.59	74 (20)	
**Hospital ward**				0.76				**0.02**
Front	216 (59.30)	55.99 ± 10.26	55 (10)		216 (59.30)	74.32 ± 14.47	71 (15.75)	
Second	148 (40.70)	58.30 ± 12.03	57 (13.75)		148 (40.70)	75.69 ± 13.16	74 (13.75)	
**Age**				**0.01**				**<0.001**
≥40	196 (53.84)	52.24 ± 21.11	53 (11.03)		196 (53.84)	72.46 ± 8.31	73 (12.25)	
<40	168 (46.16)	58.91 ± 11.06	55 (10.87)		168 (46.16)	75.34 ± 14.75	72.50 (18.50)	
**Experience work**				**0.04**				0.61
≥15	192 (52.74)	51.61 ± 47.13	53 (11.12)		192 (52.74)	71.20 ± 6.38	72 (23.5)	
<15	172 (47.25)	58.02 ± 14.04	55 (11.62)		172 (47.25)	78.12 ± 16.52	74 (17.21)	
**Gender**				**0.01**				0.41
Male	58 (15.90)	56.70 ± 11.49	54.50 (10.50)		58 (15.90)	73.12 ± 24.2	73 (17.00)	
Female	306 (84.10)	56.97 ± 10.99	56 (11)		306 (84.10)	75.12 ± 46.84	72 (9.21)	
**Marital**				0.33				0.87
Unmarried	47 (12.90)	58.12 ± 10.69	55 (13)		47 (12.90)	74 ± 12.31	73 (12)	
Married	317 (87.10)	56.75 ± 11.12	56 (11)		317 (87.10)	75.01 ± 14.19	73 (15)	
**Education**				**0.03**				0.74
Undergraduate	329 (90.40)	56.70 ± 11.17	56 (11.50)		329 (90.40)	75.02 ± 14.22	73 (15)	
Postgraduate	35 (9.60)	59.06 ± 9.86	56 (13)		35 (9.60)	73.57 ± 11.24	73 (12)	
**Overtime**				0.44				**0.007**
<20	30 (8.20)	55.20 ± 12.43	53 (12.75)		30 (8.20)	75.96 ± 14.57	75.50 (25.75)	
20–60	107 (29.40)	57.02 ± 11.01	56 (13)		107 (29.40)	79.49 ± 18.01	75 (31.00)	
60–100	162 (44.50)	57.53 ± 11.24	56 (12)		162 (44.50)	72.57 ± 10.43	71 (10)	
>100	65 (17.90)	56.09 ± 10.09	54 (9)		65 (17.90)	72.55 ± 11.92	74 (16.50)	

*SD, Standard deviation; IQR, Interquartile range. The bold values indicates P < 0.05*.

We observed a statistically significant relationship between burnout and hospital type, ward type, gender, and overtime (*P* < 0.05). Also, between the resilience of nurses and socio-demographic variables, there was a significant association regarding ward type, gender, education status, and work experience (*P* < 0.05) (shown in Table 1).

The results showed the burnout mean score of the nurses who participated in the present study was 74.88 ± 13.95, and the mean scores for emotional exhaustion, Depersonalization and Personal accomplishment were 31.4 ± 7.47, 18.25 ± 5.91, and 25.22 ± 4.67, respectively. The mean score for resilience was 56.93 ± 11.06, and the mean scores for competence, trusting, change, control, and spirituality were 17.93 ± 4.56, 14.88 ± 3.47, 12.46 ± 2.76, 6.84 ± 2.07, and 4.82 ± 1.77, respectively. Such findings highlight that, nurses had a moderate level of resilience (shown in Table 2).

**Table 2:** The mean score of burnout and resilience dimensions of participants.

	**Mean ±SD**	**Total**
**Burnout dimensions**		
Personal accomplishment	25.22 ± 4.67	74.88 ± 13.95
Depersonalization	18.25 ± 5.91	
Emotional exhaustion	31.40 ± 7.47	
**Dimensions resilience**		
Competence	17.93 ± 4.56	56.93 ± 11.06
Trusting	14.88 ± 3.47	
Change	12.46 ± 2.76	
Control	6.84 ± 2.07	
Spirituality	4.82 ± 1.77	

*SD, Standard deviation*.

The spearman correlation analysis showed that emotional exhaustion and reduced personal accomplishments were negatively correlated with total resilience (*r* = −0.442, *p* < 0.001 and *r* = −0.351, *p* = 0.03, respectively), but we did not observe a significant relation between depersonalization and resilience. In order to use the regression model to investigate the simultaneous effect of several variables on the variables of burnout and resilience and simultaneous control of some variables due to the abnormal distribution of residuals of both models, the generalized linear regression model was used (Generalized linear model). Table 3 depicts the linear regression results for the association among burnout, resilience, and socio-demographic characteristics. Results indicated that variables of hospital type, ward type, gender, and overtime were the main predictors of a high level of burnout. Also, variables of ward type, gender, education status, and work experience were the main predictors of a high level of resilience.

**Table 3:** General linear models: principal and interactive effects of demographic variable on burnout and resilience.

**Variables**	**Burnout dependent variable**			**Resilience dependent variable**		
	**β**	**SE**	***p*** **-value**	**β**	**SE**	***p*** **-value**
Type of hospital	−4.41	1.53	0.004	−1.03	1.26	0.42
Hospital ward	−1.51	1.43	0.01	−2.16	1.16	0.06
Gender	−1.56	1.99	<0.001	0.02	1.63	<0.001
Marital	−1.31	2.15	0.60	0.57	1.76	0.74
Education	1.35	2.40	0.57	−2.08	1.96	0.02
Overtimes	2.71	3.03	0.37	−1.27	2.47	0.61
Age	6.04	2.16	0.005	0.42	1.78	0.81
	−0.58	2.01	0.77	1.14	1.64	0.49
	−0.29	0.34	0.39	−0.22	0.28	0.42
Experience work	0.23	0.33	0.48	0.17	0.27	0.01

*β, Regression coefficient; SE, Standard error*.

## Discussion

This study aimed at investigating the burnout status and its relationship with resilience in nurses working in hospitals in Iran. Evidence highlights the impact of psychological stress on nurses in pandemic circumstances (27, 30). Our findings confirmed that the nurses' level of burnout was high; this is in line with the results of several earlier studies performed among healthcare providers during epidemics (31–33). According to previous studies, these findings are attributable to the physical exhaustion due to the excessive workload, shortage of staff and necessary equipment for care delivery to patients with COVID-19, death of patients, inequity, and respect to each other, different values regarding the organizations, lack of support from other organizations, working closely with infected patients for longer shifts, and fear of catching the disease or spreading it among people (5, 10, 12, 30). Also, Frontline nurses reported higher level of burnout in comparison with their co-workers in other parts. As stated in other researches, direct work with people infected with corona virus encompasses aggregated vicarious trauma for workers with direct impacts on burnout. This finding is consistent with the findings of other studies (9, 12). These findings express the need for hospitals to use protective strategies. In the pre-COVID-19 period, according to the reports, the overall prevalence of job burnout in the hospitals of Iran was 25% during 2000–2017. The highest job burnout was recorded in Tehran in 2009, 75% (34, 35), the prevalence burnout in our study was relatively higher.

High workloads of nurses can cause burnout regading emotional exhaustion, reduced personal accomplishment, and depersonalization (33). In this study, frontline nurses stated a moderate to a high level of burnout in emotional exhaustion (29.13 ± 10.30) and depersonalization (12.90 ± 4.67) and burnout was less affected concerning personal accomplishment (37.68 ± 5.17). Among the sub-dimensions of burnout among respondents, emotional exhaustion was the most commonly detected, and roughly half of them in that study experienced it. Also, it is related to heavy activities, time constraint, stressful situation, inadequet PPE, and encounter with patients infected with COVID 19. This finding is consistent with the results reported by other studies (12, 30).

Our findings highlight that nurses have a moderate or high level of psychological resilience, which is in line with findings from recent studies (33, 36, 37). Our study revealed that more COVID-19 related burnout was related to less ability to bounce back quickly in difficult periods where changes, drawbacks, obstacles, disillusions, and lack of success are likely to happen. Moreover, the links between burnout and psychological resilience were also in line with present studies (36, 37). Accordingly, when people experience high stress and harsh situations, such as the pandemic of COVID-19, resilience is the adaptation process against distressful events, and can be considered as an efficient criteria in keeping mental health (17).

The two dimensions, emotional exhaustion, and reduced personal accomplishment, had meaningful negative correlations with resilience. The obtained correlation was weak (33), but in the study conducted by Rushton et al. the association between burnout and resilience was strong (38). A negative correlation between work-related burnout was observed in other studies (39, 40). In addition, the study by Azizi and Nazemi in Iran is in line with our investigations (41). Such findings confirm that a high level of resilience can manage stress and tackle obstacles in life.

A researcher showed that younger age, marital status, sex, workload, and management issues were related to burnout among participants (42). But a study revealed a relationship between emotional exhaustion and sex and education level of the frontline nurses. Besides, depersonalization was associated with sex, age, and clinical experience. The dimension of personal accomplishments was related to age, marital status, and clinical work exposure (32).

Another factor was the level of education. In our study, participants had better levels of resilience, as they used social resources more efficiently With an increase in educational level people perceive the importance of social support resources, learn how to get access to resources, and enhance their utilization. Our findings are inconsistent with the findings of another study (14).

In addition, the current findings have revealed an association between female sex and more risk for burnout. Based on literature, females have a tendency to be more susceptible to experiencing the signs of stress particularly, nurses (43, 44). Given that nurses with direct contact with COVID-19 patients; they had to have less contact with their own families. This caused greater emotional stress and physical exhaustion (43).

Nurses with more working experience had higher mean resilience scores (45). The major reason for that was related to the fact that nurses with more working experience could balance and deal with emergencies.

## Limitations

Our study has several limitations. First, it has a cross-sectional design which limits the ability to interpret the causal relationships between the different variables in this study. Second, self-reporting has limitations regarding multiple biases. Third, the study sample was only chosen from one city. A wider geographical range is suggested. It is also recommended that future studies be conducted by using longitudinal research methods and randomized sampling.

## Conclusion

Our findings showed that during COVID-19 the prevalence of burnout among nurses was high.

Due to the importance of identifying and decreasing the burnout of nurses, they should not experience emotional exhaustion, but should enhance their personal achievements. On the other hand, resilience is a protective criterion of the mentioned signs, so it would be suggested to include the promotion of resilience in the design of interventions to reduce burnout, as other authors have mentioned. In addition, the establishment of positive working conditions and the promotion of a healthy lifestyle are moreover suggested.

As the condition of COVID-19 lasts and there is a rise in workload in a context of uncertainty and insecurity, it is expected that burnout might get worse. The remedy will need increased money support for mental health, especially for people who report symptoms of burnout. It is essential to circumvent and decrease psychological distress among nurses. Future intervention plans should include appropriate psychosocial support, stress management programs, counseling, telemedicine, and informal support groups for nurses. They are one of the foremost important assets within the battle against the COVID 19 widespread. Thus, health policymakers and managers should hold such interventions and develop situation-specific ways to enhance a healthy workplace and preventing burnout amid the COVID-19 pandemic.

## Data Availability Statement

The datasets presented in this article are not readily available because the datasets generated and/or analyzed during the current study are not publicly available due to restrictions of the Ethics Committee of Sirjan University of Medical Sciences. Requests to access the datasets should be directed to Hojjat Sheikhbardsiri, hojat.sheikhbardsiri@gmail.com.

## Ethics Statement

Ethical approval was obtained from the Ethics Committee of Sirjan School of Medical Sciences, Sirjan, Iran (ethical approval code: IR.SIRUMS.REC.1400.008). The patients/participants provided their written informed consent to participate in this study.

## Author Contributions

MJ was responsible for the analysis of the data as well as for writing the initial draft of the manuscript sections of methods and results. HS participated in the analysis of the data and design of the initial project. AK and TB were responsible for writing the initial draft of the manuscript sections of introduction and discussion. HS and AK were responsible for data collection, data cleaning, and data entry. MJ and HS were responsible for designing the project, overseeing the study, and finalizing the manuscript. All authors have read and approved the final manuscript.

## Conflict of Interest

The authors declare that the research was conducted in the absence of any commercial or financial relationships that could be construed as a potential conflict of interest.

## Publisher's Note

All claims expressed in this article are solely those of the authors and do not necessarily represent those of their affiliated organizations, or those of the publisher, the editors and the reviewers. Any product that may be evaluated in this article, or claim that may be made by its manufacturer, is not guaranteed or endorsed by the publisher.
